# Expression of the Plasma Cell Transcriptional Regulator Blimp-1 by Dark Zone Germinal Center B Cells During Periods of Proliferation

**DOI:** 10.3389/fimmu.2018.03106

**Published:** 2019-01-09

**Authors:** Daniel Radtke, Oliver Bannard

**Affiliations:** MRC Human Immunology Unit, Nuffield Department of Medicine, MRC Weatherall Institute of Molecular Medicine, University of Oxford, Oxford, United Kingdom

**Keywords:** germinal center, antibody affinity maturation, B cell, humoral immunity, differentiation

## Abstract

Long-lived plasma cells (PCs) develop in germinal centers (GCs) by the differentiation of affinity matured B cells. Antibody affinity maturation involves iterative rounds of somatic hypermutation in dark zones (DZs) and selection in light zones (LZs), however the details of where, when and how PC commitment occurs are not well-understood. Fate bifurcation at the time of selection is one possibility, with the very highest affinity GC B cells differentiating as an alternative to DZ re-entry. However, how this model fits with a need to also retain these clones in the response is not clear. Here, we show that subsets of bona fide DZ cells express the plasma cell master regulator Blimp-1 at low levels during periods of proliferation. *Ex vivo* culture experiments demonstrate that these cells are not yet committed to plasma cell differentiation but that they may be sensitized to go down that route. Contrary to models in which T cells directly select GC B cells to begin expressing Blimp-1, we found that expression of this transcriptional regulator occurred even when follicular helper T cells were ablated. We speculate that Blimp-1 may be induced during proliferation in the DZ, and that as such single selected cells might give rise to both GC and post-GC progeny.

## Introduction

Adaptive humoral immune responses against T-dependent antigens involve early waves of antibody production that are followed by the differentiation of long-lived antibody secreting plasma cells. Early antibodies are mostly made by short-lived plasmablasts that develop through extra-follicular proliferation and differentiation of activated B cells. While extrafollicular plasmablast derived antibodies may play important roles in containing and potentially clearing acute infections, their affinities are usually quite low and the cells that make them are mostly short-lived. Therefore, they are not major contributors to long-term immunity. In contrast, GC derived plasma cells are affinity matured and some have the potential to continue secreting antibodies for many years, or even the life of the host.

GCs form in the B cell follicles of secondary lymphoid tissues in the days following an infection or immunization and are sites of antibody somatic hypermutation (SHM) and selection (1–3). GCs polarize into distinct light zones (LZ) and dark zones (DZ) that GC B cells transit back and forth between many times during the course of responses. The movement of cells between the two zones is associated with changes in gene expression and behavior (4–6). LZs form proximal to sites of antigen entry and can be distinguished by the presence of specialized stromal cells known as follicular dendritic cells (FDCs) that sequester immune complexes through their high expression of complement and Fc receptors. DZs are sites of rapid proliferation and the B cells of this zone express activation induced cytidine deaminase (AID, *Aicda*) and various error prone repair pathway genes that catalyze immunoglobulin variable region gene SHM to generate new antibody variants.

GC B cells exit cell cycle in the DZ before moving to the LZ to test their freshly minted B cell receptors (BCRs) through selection events that involve the presentation of peptide-MHCII complexes to follicular helper T (Tfh) cells (5, 7). A common side product of SHM is the generation of cells with damaged immunoglobulin genes and these may be screened out before cells exit the DZ (8, 9). Affinity enhancing mutations enable LZ B cells to capture more antigen from FDCs, and this in turn allows them to present more peptide-MHC class II complexes and increases their chances of receiving help (10). Stronger T cell interactions may also drive cells to divide faster and more times upon returning to the DZ (11, 12). It is thought that only the most successful 10–30% of LZ GC B cells undergo cyclic re-entry to the DZ (2), while cells that fail this selection checkpoint mostly die by neglect (13).

Initiation of plasma cell differentiation occurs within GCs and involves induction of Blimp-1 (encoded by *Prdm1*) expression (14–16). Blimp-1 is the main plasma cell transcriptional regulator and its expression leads to the repression of various important pathways such as those that define the B cell lineage (e.g., *Pax5, Bach2*), that are required for proliferation/GC metabolism (e.g., c-*myc*), and that are needed for antigen presentation (e.g., *Ciita*) (17–19). Roles for Blimp-1 also include the direct and indirect induction of genes that facilitate large scale antibody synthesis (20, 21).

The selection criteria for plasma cell differentiation is thought to be more stringent than it is for entering the memory B cell compartment, in terms of antibody affinity. Early evidence for this came from findings that memory B cell subsets, but not plasma cell populations, are enlarged in mice expressing an anti-apoptotic Bcl2 transgene (22), and that Blimp-1^+^ GC B cell (but not memory B cell) populations are enriched for high affinity cells (23–25). Furthermore, experimental augmentation of T-B interactions leads to increased plasma cell numbers (5). Differential selection requirements fit with the proposed roles of the two different post-GC products (26). A model inferred from these observations is that high affinity LZ GC B cells may be instructed through selection cues (possibly from T cells) to undergo plasma cell differentiation as an alternative fate to DZ re-entry. Consistent with this notion, the LZ has been reported to contain small subsets of high affinity cells that might be engaged in early stages of the plasma cell differentiation program (27–29). However, how models of bifurcation during selection in the LZ might fit with a requirement for continued further maturation and expansion of the “best” clones is not known.

In this study, we investigated the context and timing of Blimp-1 expression by GC B cells. Our findings indicate that the largest “early” Blimp-1^low^ subset is one that has a DZ phenotype and that is actively dividing. DZ cells expressing Blimp-1 at low levels retained DZ-like gene expression profiles, consistent with them being a very early differentiation stage. Blimp-1 expression in the GC did not depend acutely upon signals received from follicular helper T cells, excluding the possibility that T cells directly instruct this as an alternative fate to DZ re-entry. While low Blimp-1 expression in the GC did not mark cells that are fully committed to an antibody secreting fate, such cells did show evidence of being sensitized for acquiring additional plasma cell characteristics. Our result demonstrate that DZ GC B cells may express Blimp-1 during periods proliferation, and suggest the possibility that cells that are primed or sensitized for plasma cell differentiation may emerge from that zone.

## Materials and Methods

### Mice; Immunizations, Treatments, and Infections

*Prdm1*^*wt*/*gfp*^,*Prdm1-mVenus* (Riken accession CDB0460T, http://www2.clst.riken.jp/arg/TG%20mutant%20mice%20list.html), Prdm1-yfp, Rosa26-loxP-stp-loxP-DTR, OT-II, *Rag1*^−/−^, TCRβ^−/−^ TCRδ^−/−^, and Eμ-Bcl2-22 mouse strains were described previously (15, 16, 30–36). The relevant lines were crossed to generate *Rag1*^−/−^ OTII, *Cd4*-cre Rosa26-DTR *Prdm1*^*wt*/^^gfp^ and *Prdm1*^*wt*/^^gfp^ Bcl2-tg mice. To generate mice with an ablatable cognate T cell compartment, *Rag1*^−/−^ or TCRβ^−/−^ TCRδ^−/−^ recipients were irradiated with two doses of 4.5 Gy separated by a 3 h break. Irradiated mice were reconstituted with 10% *Cd4*-cre DTR/*Prdm1*^*wt*/^^gfp^ and 90% *Rag1*^−/−^ OT-II bone marrow cells before resting for at least 8 weeks. These mice were treated with antibiotics (Baytril) from 1 week before irradiation up until they were culled. For ablation, 0.75 ug diphtheria toxin (Calbiochem) in saline was delivered by intraperitoneal (i.p.) injection. Immunizations were performed by i.p. injection of 2 × 10^8^ SRBCs i.p. (Thermo Fisher Scientific). For CD40L blockade, 0.5 mg anti-mouse CD40L (clone MR1, Biolegend), or 0.5 mg purified armenian hamster IgG control antibody (BioXcell, BE0091), was administered by i.v. injection. Intranasal influenza infections were performed under isoflurane anesthesia with 2 × 10^4^ PFU HKx/31 (H3N2) virus in 30 μl volume. Some experiments were performed using C57BL/6 mice that had been irradiated and reconstituted with bone marrow from *Prdm1*^*wt*/*gfp*^ mice. Similarly, all Blimp-1-mVenus and Blimp-1-YFP experiments were performed using WT host/transgenic donor bone marrow chimeras. For DNA labeling experiments, mice received a single i.p. injection of 1 mg EdU at the indicated time points before tissue harvest. Animals were housed in specific pathogen-free enclosures at the University of Oxford Biomedical Sciences facility. All animal experiments were approved by a project license granted by the UK Home Office and were also approved by the Institutional Animal Ethics Committee Review Board at the University of Oxford.

### Flow Cytometry

Single cell suspensions were generated using 70 or 100 μm cell strainers (BD Pharmingen). Cells were treated with Fc-receptor blocking antibody (anti-CD16/32) and stained with fluorophore coupled antibodies. In some cases, cells were fixed and permeabilised with Cytofix/Cytoperm (BD Pharmingen) before analysis. A list of antibodies can be found in Table S3. For DNA content or intracellular staining, cells were stained with antibodies to surface antigens before fix/perm. Perm steps were usually performed overnight in 3–5 ml volumes. DAPI staining was performed at a concentration of 2 μg/ml DAPI that was added just before analysis. Samples were measured or sorted on BD LSR Fortessa X20, LSRII, FACSAria IIIu flow cytometric analysers. Live sort experiments were performed using 100 or 130 uM nozzles. Data was analyzed using FlowJo software (Tree Star).

### Histology

Tissues were fixed in 4% PFA/PBS for 2 h at 4°C, washed three or more times and then transferred successively to 10, 20, and 30% sucrose/PBS with 30 min incubation at each step except the last which was overnight. Tissue was snap frozen in OCT embedding medium (Thermo Scientific). Thirty micrometer sections were cut, dried and blocked (PBS with 0.3% TritonX100, 0.2% BSA, 0.1% NaN3, and 3–5% relevant serums). GFP was detected using a rabbit anti-GFP antibody followed by donkey anti-rabbit AF488. A full list of the antibodies used is included in Table S3. Staining steps were performed for >12 h using the same blocking solution as is listed above. Slides were mounted in ProLong Diamond Anti-fade Mounting reagent (Life Tech.) and images were taken with a Zeiss 780 Inverted or a Zeiss 780 Upright MP confocal microscope using a 20 × oil immersion objective. Imaris software (bitplane) was used for analysis and processing. For EdU stainings, the Click-iT Plus EdU Alexa Fluor 555 Imaging Kit (Life Tech) was used according to manufacturer's instructions. The EdU stain was performed after blocking but before antibody staining.

### *Ighv* Sequencing and Analysis

Reverse transcription and PCR amplification were performed according to a published protocol (37). Briefly, single cells were sorted into a 96 well PCR plate with 10 μl of capture buffer made up of 5 ml RNAse-free water (Ambion), 50 μl 1M Tris pH 8.0 (Gibco), 125 μl RNasin (Promega) and frozen at −80°C. Reverse transcription after defrosting was performed using the Maxima cDNA Synthesis Kit (Thermo Fisher Scientific). A mix containing 3 μl 5 × buffer mix, 1.5 μl Maxima Enzyme mix and 1.5 μl 5% IGEPAL (Sigma-Aldrich) was added. For a one step pre-amplification of variable heavy chain regions, the MsVHE primer that is capable to amplify most heavy chain variants combined with specific primers for IgG1, IgG2b, IgG2c, IgA, and IgM isotypes was used. Successful amplification was confirmed on a diagnostic gel and 5 μl PCR product of amplified samples were cleaned up for sequencing by adding 0.5 μl ExoI and 1 μl rSAP (New England Biolabs) for 15 min at 37°C followed by 15 min at 80°C for heat inactivation. Samples were Sanger sequenced with the MsVHE primer. SeqTrace (0.9.0) was used to generate FASTA files from chromatograms (38). In the program the minimal confidence score to define a base was set to 30 and the ends were trimmed automatically until eight out of ten bases passed the quality score. Bases that failed the quality score in the trimmed sequence were assumed to be the most likely base assigned by SeqTrace when no quality score was set for alignment. Sequences were assigned to variable (V), diversity (D) and joining (J) gene segments using IgBlast (39). Only sequences with “V-J” regions in frame and no early stop codons were further analyzed and only somatic mutations at positions with a SeqTrace quality score of 30 or better were counted. Custom R scripts were used to assign mutations to quality scores and the FlowCore package (R package version 1.42.3) was used to combine flow data from the sort with mutation data (40).

### RNA Sequencing

RNA libraries to sequence 100 cells were prepared using an adapted Smart-seq2 protocol (41). Cells were sorted directly into 8 μl lysis mix. Reaction volume was doubled relative to Picelli et al. from reverse transcription to pre-amplification to aid FACS stream alignment into 0.2 ml tubes. Normal volumes were used at all stages following the pre-amplification steps. Seventeen or eighteen cycles of PCR amplification were used for GC and follicular samples, respectively. cDNA purification was performed using Ampure XP beads (Beckman Coulter) at a sample:bead ratio of 1.7. Libraries were analyzed with a High Sensitivity Analyser (Agilent) and cDNA tagmentation was performed with the Nextera XT DNA Sample Preparation kit (Illumina). Libraries were quantified using PicoGreen (Illumina), sized using the High Sensitivity Analyser and equal amounts of tagmented cDNA from each library were pooled. Sequencing was performed on an Illumina NextSeq500 using FC-404-2005 NextSeq 500/550 High Output Kits v2 (75 cycles).

For data analysis, adapters were trimmed with Trim Galore (0.4.1) and reads were aligned with STAR (2.5.3a) (42) to the *Mus Musculus* genome build GRCm38/mm10. FeatureCounts was used to generate reads per gene (43). Further analysis was performed in R using limma (44) and EdgeR (45, 46) according to published pipeline with minor adaptions (47). For name conversions between Entrez gene IDs and Gene Symbols the Mus.Musculus package was used and if duplicated gene names were present the first occurrence was kept while the others were deleted. EdgeR functions were used to calculate kilobase of transcript length per million mapped reads (RPKM), counts per million (CPM) or log2-CPM. Genes expressed at 1 cpm or higher in at least 3 samples were kept for further analysis and a normalization for library size was performed. Unsupervised clustering was performed based on the thousand genes with the largest standard deviation between samples using limmas “plotMDS” function and the first two dimensions explaining the highest proportions of variation were plotted. Library size normalized log2-CPM data was precision weighted using limmas “voom” function. Then differential expression was analyzed using a linear model in limma and Benjamini-Hochberg (BH) adjusted *P*-values were used to determine significance. For heatmaps, log_2_ transformed mean RPKM values for biological replicates were calculated and negative values were set back to zero before values were scaled and heatmaps were drawn with the gplots package. Only a fraction of DZ and LZ signature genes from Victora et al. were used to draw corresponding heatmaps (48). Genes were selected to have an adj. p-val. between DZ^neg^ vs. LZ^neg^ < 0.05 and had to be up in LZ^neg^ vs. DZ^neg^ for LZ signature genes or up in DZ^neg^ vs. LZ^neg^ for DZ signature genes. For the top differentially expressed gene heatmap, only genes with an adj. *p* < 0.05, a fold change >2.5 and a mean expression >5 RPKM in at least one of the CD138^−^ GC populations were chosen. For expression scatter plots log_2_ transformed mean RPKM values were calculated for biological replicates and negative values were set to zero. For gene set enrichment analysis, GSEA (3.0) from the Broad Institute was used following the recommended instructions (49).

### Single Cell Gene Expression Analysis

Single cells were sorted into 96 well plates with 5 μl one-step mix for cDNA reverse transcription and specific gene amplification for target genes (Table S3). The mix contained 2.5 μl 1 × Cells Direct Reaction Mix from the Cells Direct One-Step qRT-PCR kit (Life Tech.), 0.05 μl SUPERase-In RNase Inhibitor (Ambion), 0.6 μl RT-Taq mix, 0.6 μl Tris-EDTA (TE) (Life Tech.) and 200 nM primers. The cDNA was pre-amplified in 22 cycles and diluted to 25 μl volume with Tris-EDTA. For quantitative PCR 1.8 μl sample were prepared and loaded/run on a Biomark 192.24 Dynamic Array IFC (Fluidigm) according to manufacturer's instructions using TaqMan Universal PCR Master Mix (2X) (Life Technologies). BioMark Real-Time PCR Analysis Software and R were used for analysis. Cells that expressed fewer than 10 of 24 analyzed genes (not all shown) were discarded from analysis as assumed the reaction low quality (Ct cut-off value of 30). For co-expression analysis of *Pax5, Aicda*, and *Prdm1* cells were counted as positive for a gene if they gave a Ct value of <25.

### Single Cell and Small Population “Nojima” Cultures

Single cells “Nojima” cultures were performed as previously described with only minor modifications (50). Briefly, NB21 feeder cells were seeded at a density of 1,500 cells per well into a flat bottom 96 well plate the day before sorting in 100 ul medium. The following morning, a further 100 ul medium was added with IL-4 (Peprotech) to provide final concentration of 2 ng/ml. Culture medium was the same as in the published protocol except the FBS was from Life Tech (Cat. 10500056). Cells were sorted directly into culture wells and incubated at 37°C and 5% CO_2_. In these experiments, GC B cells were sorted using an alternative gating scheme (B220^+^ CD38^low^ IgD^low^ GL7^+^) to avoid using antibodies against the death receptor, CD95. From day 2, culture media volume was increased to 300 ul and half volume was replaced daily without additional IL-4. Cells were harvested on day 9 and stained with CD19 and CD138 antibodies before fixation (4% PFA) and FACS analysis. Cell numbers were calculated by adding 123 count eBeads (eBioscience) at the time of facs staining. Conditions were the same for 500 cell cultures except that analysis was performed at 48 h and no fixation was performed.

### Statistical Analysis

Statistical analysis was performed using Prism 7 (GraphPad) except for RNA sequencing described above. Statistical tests used are given in figure legends.

## Results

### The Plasma Cell Master Regulator Blimp-1 Is Expressed at Low Levels by a Subset of Dark Zone Cells

**Figure 1:**
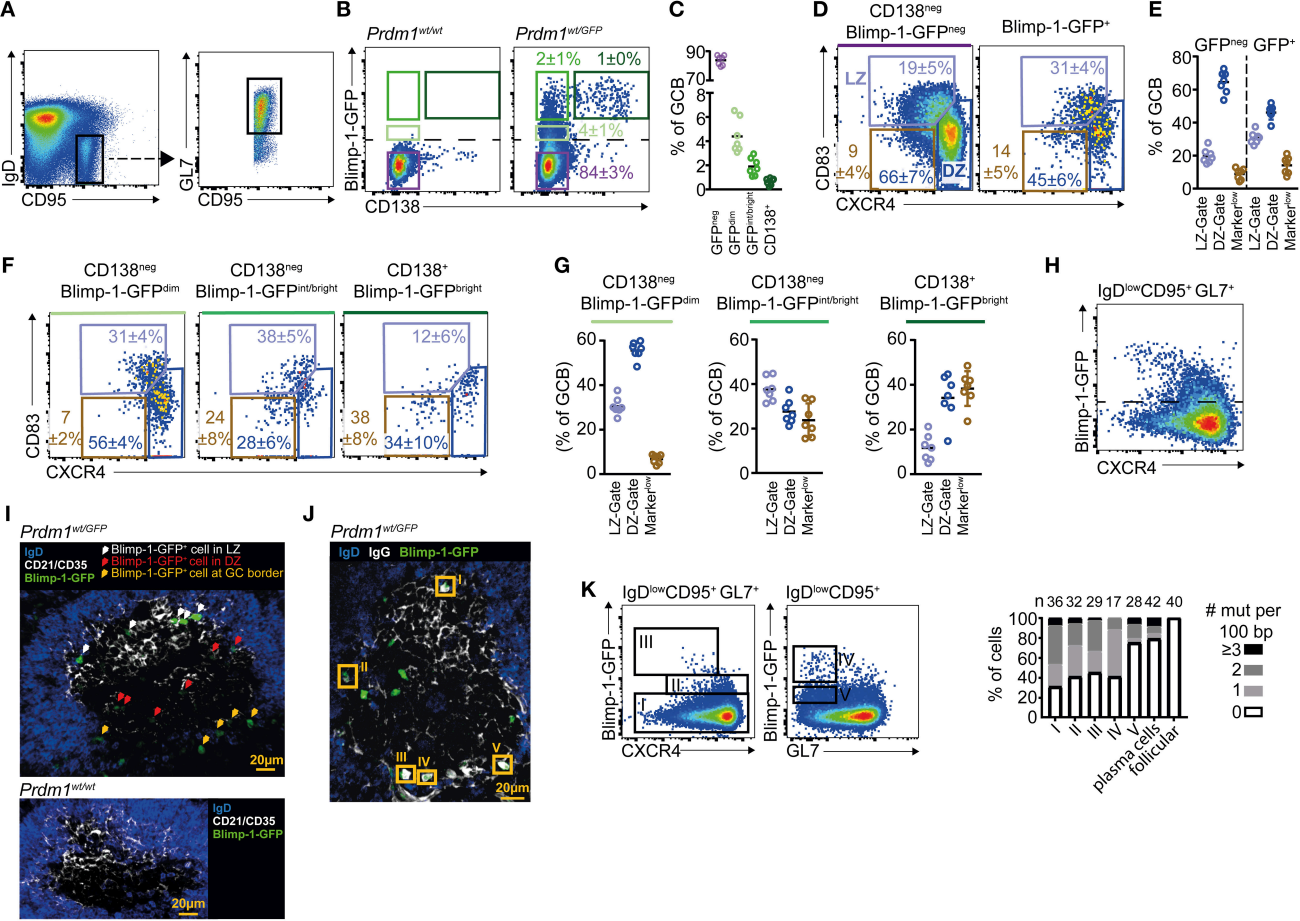
A Blimp-1-GFP^dim^ population is found in the germinal center dark zone. *Prdm1*^*wt*/*gfp*^ mice were analyzed by FACs at day 10 after SRBC immunization. **(A)** Splenic GC B cells were gated as B220^+^IgD^low^CD95^+^GL7^+^. **(B)** Pre-plasma cell differentiation stages were defined based upon Blimp-1-GFP and CD138 levels. The dashed line marks the detection threshold for GFP^+^ cells. **(C)** Quantification of populations defined in **(B)** using the same color code. **(D)** Frequencies of LZ (CXCR4^low^ CD83^high^) and DZ (CXCR4^high^ CD83^low^) cells among Blimp-1-GFP^neg^ and Blimp-1-GFP^pos^ GC B cells. A further gate is shown around cells falling outside of DZ/LZ gates (CXCR4^low/neg^ and CD83^low/neg^). **(E)** Summary of results as in **(D)**, from multiple mice and experiments. **(F)** DZ/LZ frequencies were determined for the different differentiation stages defined in **(B)**. **(G)** Summary of results as in **(F)**, from multiple mice and experiments. **(H)** CXCR4 vs. Blimp-1-GFP on total GC B cells. **(I)** Confocal image of a splenic GC in which Blimp-1-GFP^+^ cells are identified and marked by arrows, with colors indicating their position. CD21/35 demarcates the LZ. **(J)** Confocal image in which Blimp-1-GFP^+^ cells with high intra-IgG levels are highlighted by orange rectangles. **(K)** Mutation frequency per 100bp of *Ighv* sequence was determined for the indicated populations I-V. Results for follicular B cells (IgD^neg^ CD95^neg^ GL7^neg^) and plasma cells (B220^int^ Blimp-1-GFP^bright^ CD138^+)^ are also shown. Plots in **(A,B,D,F)** and **(H)** are representative of at least 7 mice from 3 experiments. Numbers shown are percent in gates, ± S.D. For confocal images, varying numbers of GFP^+^ cells were detected per GC. GC localized Blimp-1-GFP^+^ cells were detected in 11 separate mice from 2 experiments **(I)** or 7 mice from 3 experiments **(J)**. Data in H is from two independent experiments but population V was only included in one.

To begin exploring the context in which GC B cells initiate plasma cell differentiation, we examined Blimp-1 expression by GC B cells using a GFP transcriptional reporter mouse line (*Prdm1*^wt/gfp^ heterozygous mice) (15). Mice were immunized by i.p. injection with sheep red blood cells (SRBCs) and IgD^low^ CD95^+^ GL7^+^ splenic GC B cells were gated on day 10 (Figure 1A). GFP detection thresholds were set using GC B cells from GFP^negative(neg)^ wild type (WT) mice. As expected (14, 16, 23, 28), small but distinct GC B cell subsets were Blimp-1-GFP^+^ in the reporter mice (Figure 1B). The fluorescence intensities for individual cells differed over the range of approximately one and a half to two logs, presumably reflecting differences in duration that the gene had been expressed for and the specific stage of the differentiation process (15, 16). The plasma cell marker CD138 (syndecan-1) was mostly restricted to cells with higher GFP levels (Figures 1B,C). Back-gating revealed CD138^+^ Blimp-1-GFP^bright^ cells as having slightly lower GL7 levels relative to the total GC B cell population, as might be expected for later differentiation stages (Figure S1A). Therefore, IgD^low^ CD95^+^ GL7^+^ GC B cell gates contain cells at different phases of plasma cell commitment, and the combination of Blimp-1 and CD138 detection/staining provided a means to gate likely “early,” “mid,” and “late” expressing cells.

GC B cells undergo SHM and cellular division in the DZ state before transitioning to their LZ phase for selection (1, 3). DZ and LZ cells are distinguished by their differential expression of the transmembrane proteins CXCR4, CD83, and CD86, with the former subset being CXCR4^high^, CD83^low^, and CD86^low^ (4, 5, 48). We therefore compared staining patterns for these proteins on Blimp-1-GFP^neg^ and Blimp-1-GFP^+^ GC B cells (Figures 1D,E). Models in which cells bifurcate to begin expressing Blimp-1 immediately following selection as an alternative to cyclic re-entry predict that “early” CD138^neg^ Blimp-1-GFP^dim^ GC B cells should mostly have a LZ phenotype. However, GC B populations with this “early” phenotype were only modestly enriched for CXCR4^low^ CD83^+^ LZ cells (31 vs. 19% among Blimp-1-GFP^neg^ cells). Instead, the majority (56%) of these cells had a CXCR4^high^ CD83^low^ DZ phenotype (Figures 1F,G). Similar findings were made using CXCR4 vs. CD86 LZ/DZ gating schemes (Figure S1B). Interestingly, within “mid” CD138^neg^ Blimp-1-GFP^int/bright^ and “late” CD138^+^ Blimp-1-GFP^bright^ gated populations, the frequencies of DZ phenotype cells was lower but this was mostly a result of there being increased frequencies of cells that fall outside of classical LZ/DZ gates (referred to as “Marker^low^” in Figure 1).

Due to concerns that reduced *Prdm1* transcript abundance in heterozygous gene targeted mice may impact differentiation kinetics, we performed similar experiments using Blimp-1-mVenus and Blimp-1-YFP BAC transgenic reporter mouse lines (16, 36) (Figures S1C–F). However, as in the *Prdm1*^wt/gfp^ animals, the majority of “early” CD138^neg^ Blimp-1^dim^ GC B cells in the BAC transgenic lines had a DZ phenotype (62% in Blimp-1-mVenus mice, 66% in Blimp-1-YFP mice). GC B cells from the GFP (gene targeted) and BAC transgenic mouse lines did seemingly differ in terms of their CXCR4 levels on “later” (reporter bright) cells, however, for unknown reasons. CXCR4 levels were intermediate/high on mVenus^bright^/YFP^bright^ GC B cells but were low on equivalent populations in the GFP gene targetted animals (Figure 1H and Figures S1D,F). Importantly, a correlation between low Blimp-1-GFP levels and a DZ phenotype also held up in the context of an antiviral (H3N2, HKx/31 influenza A) GC response in a different lymphoid tissue (mediastinal lymph nodes) (Figures S1G,H), and using different GC gating schemes (IgD^low^ CD95^+^ PNA^+^ and IgD^low^ CD95^+^ EphrinB1^+^) (Figures S1I,J), confirming that these observations were not specific to a specific immunization, to the spleen, or to a single GC marker.

We next made efforts to understand where Blimp-1 is expressed by GC B cells *in situ*. Splenic sections from immunized *Prdm1*^*wt*/*gfp*^ mice were immuno-stained and examined by confocal microscopy. GC LZs and DZs were distinguished by the presence or absence of FDC associated CD21/35 (Figure 1I) or IgG (immune complex) staining (Figure 1J). Comparisons to control sections from WT mice confirmed the specificity of the anti-GFP stain (Figure 1I). GFP levels in the “early” GC subsets of interest are very low and this impeded efforts to perform a careful quantitative analysis, however Blimp-1-GFP^dim^ cells were found in both zones as expected from the FACS. Blimp-1-GFP^bright^ cells were commonly, but not exclusively, found to be within the network of FDCs that demarcate the LZ, or at the very far base of the DZ, consistent with previous reports (28, 29, 51). Cells at the base of the GC were often so “deep” that it was not possible to definitively determine whether they were in or just outside the GC perimeter. Regardless of their site, Blimp-1-GFP^bright^ cells commonly contained large quantities of intracellular IgG, further confirming that they are at a late differentiation stage (Figure 1J). Individual GCs differed in the number of Blimp-1-GFP^+^ cells they contained (panel of examples show in Figure S1K), however whether this reflects true heterogeneity in plasma cell output, or instead reflects sampling noise, was not clear.

As a final effort to validate that we were analyzing true GC derived cells in our FACs experiments, single cells were sorted and their *Ighv* region genes PCR amplified, sequenced and compared to their predicted unmutated common ancestors (37). For feasibility issues, Blimp-1-expressing GC B cell populations were divided into just dim and bright subsets in these experiments. Consistent with their proposed origin, most Blimp-1-GFP^dim^ (subset II) and GFP^bright^ (subset III) GC B cells carried somatic mutation loads that were similar to that of their non-Blimp-1 expressing counterparts (subset I) (Figure 1K). Interestingly, IgD^low^ CD95^+^ cells that were Blimp-1-GFP^bright^ but had low or negligible GL7 levels (subset IV) also carried GC-like somatic mutations loads, indicating that they are GC or post GC cells in the process of reducing their GL7 levels. In contrast, IgD^low^ CD95^+^ GL7^neg^ Blimp-1-GFP^dim^ B cells (subset V) and B220^int^ CD138^+^ Blimp1-GFP^+^ plasma cells mostly were not somatically mutated. Therefore, the former population probably represents cells engaged in the extrafollicular response and the latter subset mostly contains cells that developed early in the response or via that same route.

In summary, Blimp-1 is expressed at a range of levels in the GC. CD138^neg^ Blimp-1-GFP^dim^ cells are found in both zonal subsets, but the majority of these “early” Blimp-1 expressing cells have a DZ phenotype.

### Dark Zone Cells With Low Blimp-1-GFP Levels Have Transcriptional Signatures Consistent With Their Phenotype

We extended our investigations of Blimp-1 expression in the GC beyond correlations with cell surface markers. We were conscious that plasma cells, like DZ cells, depend upon CXCR4 for their proper positioning (52), and that they may downregulate CD83 and CD86 as they differentiate, which could lead to the former being assigned as the latter. Therefore, the transcriptomes of DZ and LZ GC B cells expressing the Blimp-1-GFP reporter were determined. Cells were FACS sorted using the gates described in Figures 1B,F, with the exception that the “late” CD138^+^ Blimp-1-GFP^bright^ population was not subdivided using LZ/DZ markers. RNA sequencing (RNA-seq) libraries were prepared from 100 cell samples using the “smart-seq2” protocol (41). mRNA from follicular B cells and B220^int^ GL7^neg^ CD138^+^ Blimp-1-GFP^bright^ plasma cells was also sequenced, for comparison.

**Figure 2:**
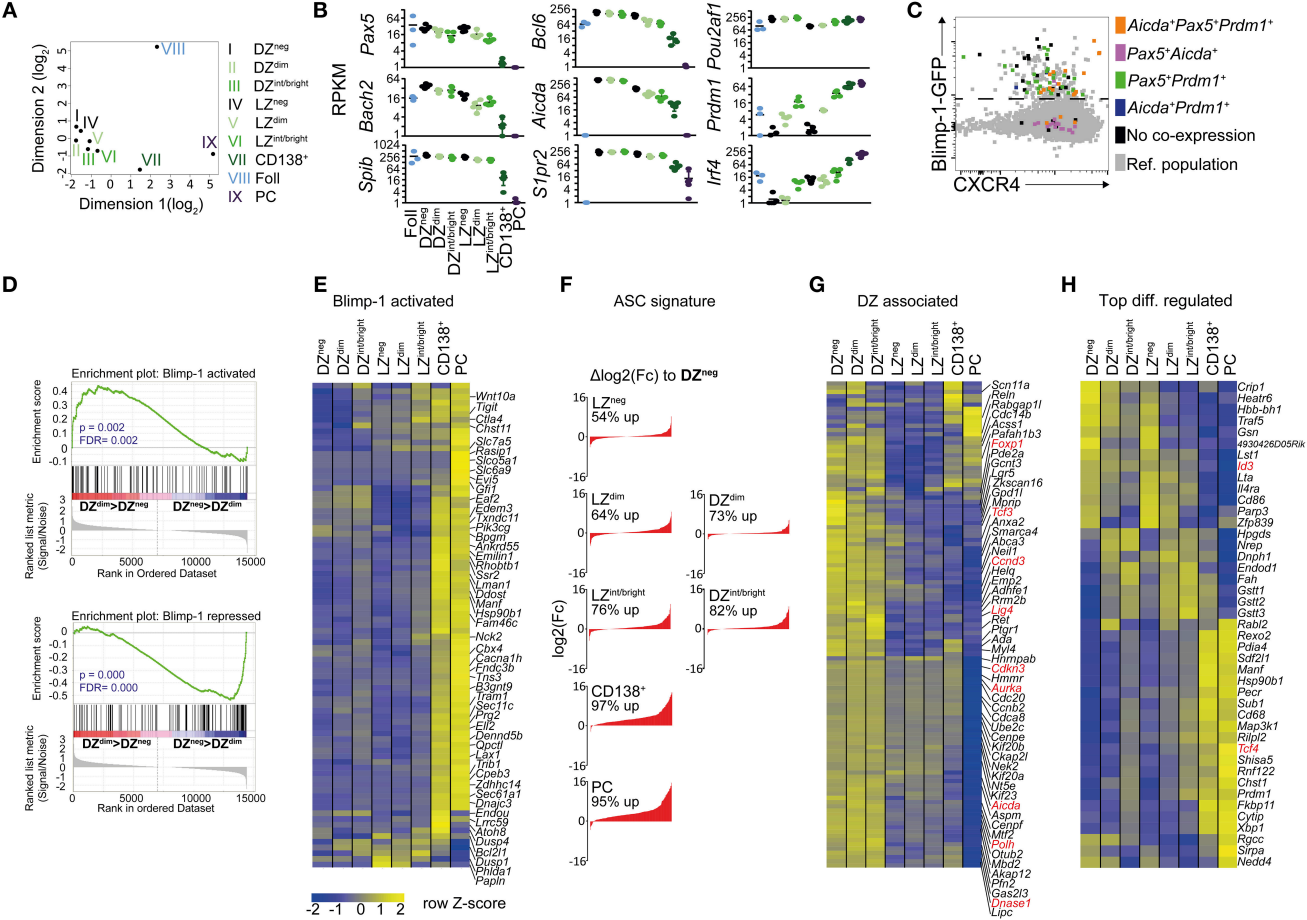
RNA-seq analysis reveals Blimp-1 is expressed by GC B cells while they still retain dark zone and light zone signatures. One hundred cell populations were sorted according to the gating scheme presented in Figures 1A–D but with slightly increased stringency on the LZ/DZ gates to ensure purity. Their transcriptomes were determined by RNA-seq. Sample names have been shortened, e.g., DZ CD138^neg^ Blimp-1-GFP^dim^ cells are referred to as DZ^dim^ in the figure. **(A)** A multidimensional scaling plot showing differences between samples based on top 1,000 genes with the largest standard deviations between samples. **(B)** log2 RPKM values for various important B cell lineage, GC regulating or plasma cell associated genes. **(C)** Single cell RT-PCR was performed on index-sorted GC B cells to confirm co-expression of the indicated genes (colored according to legend). Cells not co-expressing at least two of the indicated genes (*Aicda, Pax5, Prdm1*) are depicted in black. CXCR4 and GFP protein intensities are also indicated on the x and y axis. **(D)** Gene set enrichment analysis (GSEA) of Blimp-1 activated or Blimp-1 repressed gene sets comparing DZ^dim^ and DZ^neg^ cells. The nominal *p*-values (p) and FDR *q*-values (FDR) are given. **(E)** Relative expression levels of Blimp-1 directly activated genes. **(F)** Changes in ASC-related gene expression sorted by log2 fold difference relative to Blimp-1-GFP^neg^ DZ cells. Frequencies represent percent of ASC-genes that are increased in the indicated subset. **(G)** Differences in DZ-associated genes. Genes discussed in text are shown in red. **(H)** The top 10 most up-regulated and 10 down-regulated genes between LZ vs. any LZ-like Blimp-1-GFP^+^ population and DZ vs. any DZ-like Blimp-1-GFP^+^ population. Additional genes of interest were added to the list (depicted in red). RNAseq was performed using 5 biological replicates (mice) per group except for follicular B cells where 3 were used. Means are shown in **(E,G,H)**.

Multi-dimensional clustering analysis identified all the CD138^neg^ Blimp1-GFP expressing GC B cell populations (Blimp-1-GFP^dim^ and Blimp-1-GFP^int/bright^ cells, with both LZ and DZ surface phenotypes) as being more closely related to Blimp-1-GFP^neg^ GC B cells than they were to terminally differentiated plasma cells, confirming their “early” and “mid” differentiation statuses (Figure 2A). Consistent with their “later” phenotype, CD138^+^ Blimp-1-GFP^bright^ gated cells fell somewhere between GC B and plasma cells in this analysis.

Despite their having initiated expression of the plasma cell master regulator, DZ and LZ CD138^neg^ Blimp-1-GFP^dim^ GC B cells still expressed various important GC regulating genes such as *Bcl6, Aicda*, and *S1pr2* at levels similar to that of their Blimp-1-GFP^neg^ counterparts (Figure 2B and Table S1). Furthermore, these cells showed only modest evidence of having started downregulating expression of the B cell lineage defining genes *Pax5* and *Bach2* that are known targets for Blimp-1 mediated repression (53). Pax5 and Bach2 transcript reductions were also only marginal in CD138^neg^ Blimp-1-GFP^int/bright^ GC cells, with larger decreases being evident only at the CD138^+^ Blimp-1-GFP^bright^ stage. Surprisingly, *Irf4* mRNA levels were not raised at the “early” CD138^neg^ Blimp-1-GFP^dim^ stages, despite its high and sustained levels being one way that plasma cell differentiation is promoted (Figure 2B and Figure S2A) (54). However, *Irf4* transcripts were more abundant by the time cells reached the CD138^neg^ Blimp-1-GFP^int/bright^ stage (2 and 7-fold in LZ and DZ, respectively), and they were further increased in CD138^+^ Blimp-1-GFP^bright^ cells (2.5 and 6-fold further). Single cell RT-PCR analysis confirmed mRNAs encoding *Prdm1, Pax5, and Aicda* were present in the same cells, ruling out concerns we had about possible pooling effects (Figure 2C). Despite these results, gene set enrichment analysis (GSEA), comparing to a dataset of genes that are Blimp-1 bound and regulated in *in vitro* generated pre-plasma blasts (21), provided evidence that Blimp-1 protein was probably present and active already even in CD138^neg^ Blimp-1-GFP^dim^ cells (Figure 2D). Genes from both Blimp-1 regulated and antibody secreting cell (ASC) associated genesets (55), however, showed far greater expression changes at the “late” CD138^+^ Blimp-1-GFP^bright^ stage (Figures 2E,F and Table S1).

We next interrogated the RNA-seq datasets for evidence as to whether similarities between Blimp-1-expressing and non-expressing “DZ” cells extended beyond just surface markers. Expression of DZ signature genes (5, 48) was remarkably similar in CD138^neg^ Blimp-1-GFP^dim^ and Blimp-1-GFP^neg^ DZ populations, indicating that shared transcriptional programs were in place (Figure 2G and Table S1). For example, cells in both populations expressed cell cycle related genes (e.g. *Ccnd3, Aurka, Cdkn3*), DNA editing genes (e.g., *Aicda, Polh, Lig4, DnaseI*), and transcription regulating genes (e.g., *FoxP1, Tcf3*) at levels above that of LZ cells. Continued expression of many DZ signature genes was evident even in “mid” CD138^neg^ Blimp1-GFP^int/bright^ cells, and *Aicda* transcripts were still detected even in the “late” CD138^+^ Blimp-1-GFP^bright^ subset (albeit an approx. 4-fold and 2-fold reduced level relative to in DZ and LZ Blimp-1-GFP^neg^ cells, respectively). The detection in Blimp1-expressing cells of mRNAs encoding DNA editing genes was surprising as it raises the possibility of continued SHM after Blimp-1 expression, however regulation of the SHM machinery may occur in a post-transcriptional manner at this phase. Importantly, Blimp-1-expressing DZ cells, in contrast to LZ-like populations, contained DZ-like levels of most LZ signature (5, 48) mRNAs such as *Fcer2a, Cd40, Nfkbia, and Gpr18*3 (Figure S2A). The “early” Blimp-1 expressing LZ subset also displayed a transcriptional profile that was very similar to its non-Blimp-1 expressing counterpart (Figure 2G and Figure S2A). Consequently, Blimp-1 is expressed at low levels in DZ and LZ cells at times when they retain mRNAs encoding important GC and subset regulating genes.

A further goal of the RNA-seq experiments was to identify transcriptional changes occurring early after Blimp1-expression in GC B cells. We therefore compiled a list of the top 10 genes whose expression was increased or decreased in “early” CD138^neg^ Blimp-1-GFP^dim^ and “mid” CD138^neg^ Blimp-1-GFP^int/bright^ DZ populations, relative to their non-GFP expressing counterparts (Figure 2H). Similar comparisons for the LZ subsets were also performed, with the resulting heat map showing gene expression patterns for all populations. In addition, we identified genes whose expression was changed already in the “early” Blimp1-expressing subsets (CD138^neg^ Blimp-1-GFP^dim^ cells) and maintained in CD138^neg^ Blimp-1-GFP^int/bright^ cells. Of 53 genes identified as being differentially expressed in CD138^neg^ Blimp-1-GFP^dim^ DZ cells, 47 remained changed in the CD138^neg^ Blimp-1-GFP^int/bright^ subset. In LZ cells, 57 of the genes that differed in CD138^neg^ Blimp1-GFP^dim^ cells also were changed in CD138^neg^ Blimp-1-GFP^int/bright^ cells (Figure S2B). Surprisingly, of the 104 differentially expressed genes identified by this analysis, only 9 were shared between the DZ and the LZ subsets.

In summary, these findings confirm that subsets of bona-fide DZ and LZ cells express low levels of the plasma cell master regulator Blimp-1, but that most plasma cell associated gene expression changes occur only at later stages of pre-plasma cell development.

### Rapid Proliferation Associated With Blimp-1 Expression by Dark Zone Cells

**Figure 3:**
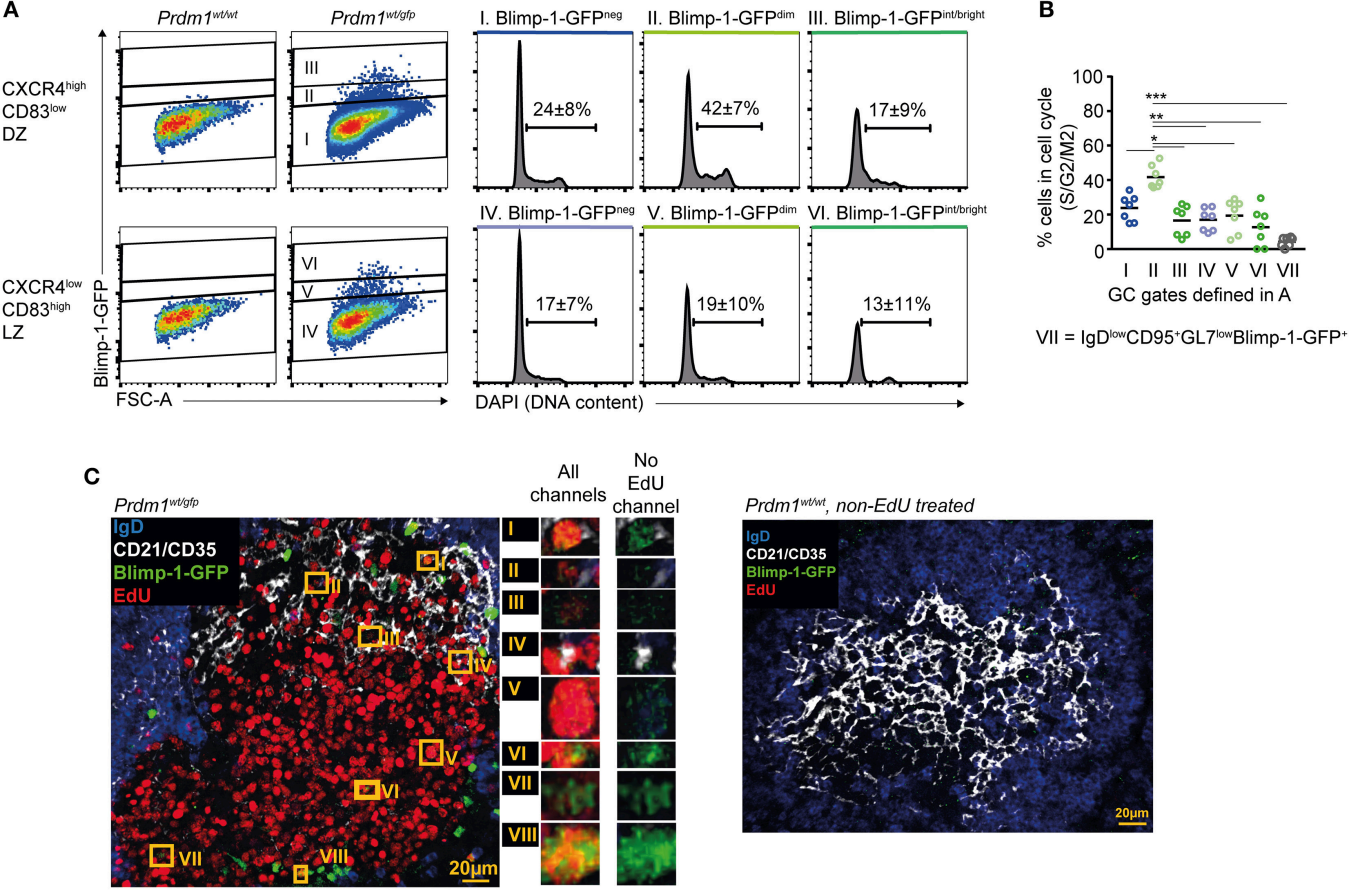
Blimp-1 is expressed at low levels during periods of proliferation. **(A)** DNA content based cell cycle analysis was performed on the indicated splenic GC subsets from *Prdm1*^*wt*/*gfp*^ mice on day 10 following SRBC immunization (left). Frequency means (±S.D.) of cells in S/G2/M are indicated on histograms. **(B)** Summary of results as in **(A)**, from multiple mice and experiments. **(C)** Confocal micrographs showing EdU incorporation by Blimp-1-GFP^+^ GC B cells (left). Mice received single i.p. injections of EdU 5 h before analysis. Blimp-1-GFP^+^EdU^+^ cells in the GC area are highlighted by orange rectangles and are shown in magnification with or without the EdU channel on. A *Prdm1*^wt/wt^ non-EdU treated control sample is also shown (right). For **(B)** a Kruskal-Wallis test with Dunn's test was performed (*n* = 7; pooled from 3 independent experiments). Horizontal lines indicate means *P < 0.05; **P < 0.01; ***P < 0.001. **(C)** Is representative of GCs from 4 mice.

The finding that Blimp-1 may be expressed in DZ GC B cells raised the question of whether clonal expansion continues during or after its expression is initiated. Extrafollicular antibody responses involve periods of pre-plasmablast and/or plasmablast proliferation (56), but whether similar events occur in established GCs has not been investigated. Splenocytes from immunized *Prdm1*^wt/gfp^ mice were fixed and their DNA content was determined by DAPI staining. This procedure caused significant losses in GFP fluorescence intensities that led the Blimp-1-GFP^dimmest^ cells to appear Blimp-1-GFP^neg^, however we were still able to identify cells with a range of GFP intensities (Figure 3A). A greater frequency of DZ cells (Blimp-1-GFP^neg^) were actively dividing than were LZ cells (24 vs. 17%), as expected (Figures 3A,B) (4, 5). Surprisingly, the frequency of proliferating DZ cells was even higher for the Blimp-1-GFP^dim^ subset (42 vs. 24% for Blimp-1GFP^dim^ DZ and Blimp-1-GFP^neg^ DZ, respectively), indicating that these are among the most active cells in terms of cellular division. Similar findings were made using just CXCR4 to gate DZ cells (Figure S3A), and when the analysis was performed at very early (day 6) or late (day 16) stages of the response (Figures S3B,C). An inverse correlation existed between GFP florescence intensity levels and the likelihood of a cell undergoing division, consistent with Blimp-1-GFP^bright^ cells representing later differentiation stages (Figures 3A,B and Figure S3A). The presence of proliferating Blimp-1-GFP^+^ cells within GC structures was confirmed through immunostaining and confocal microscopy of splenic sections from mice that received EdU during the preceding 5 h (Figure 3C and Figure S3D). We unfortunately failed, however, to perform a more quantitative *in situ* analysis because GFP detection sensitivity was seemingly negatively impacted by development steps for EdU analysis.

These results demonstrate that Blimp-1 expression is induced during, or maintained by, DZ GC B cells during periods of proliferation.

### Dark Zone GC B Cells Retain the Capacity for Extensive Clonal Expansion After Initiating Blimp-1 Expression

The finding that Blimp-1 expression occurs concordantly with cellular division in DZ cells led us to next ask whether Blimp-1^+^ GC B cells are all committed to quickly differentiate to a post-proliferative state, or whether instead some cells may retain the capacity for extensive clonal expansion. To test this, we made use of the “Nojima” culture method (50), a novel single cell GC B cell feeder assay developed by Kuraoka et al. that involves FACS sorting single GC B cells into tissue culture wells containing CD40L and Baff expressing “NB21” feeder cells (in the presence of exogenous IL-4), followed by *in vitro* incubation for 9 days. By performing indexed FACS sorts (where staining profile information is retained for each cell/well), we were able to correlate proliferative potential to the phenotype of cells at the time of plating.

**Figure 4:**
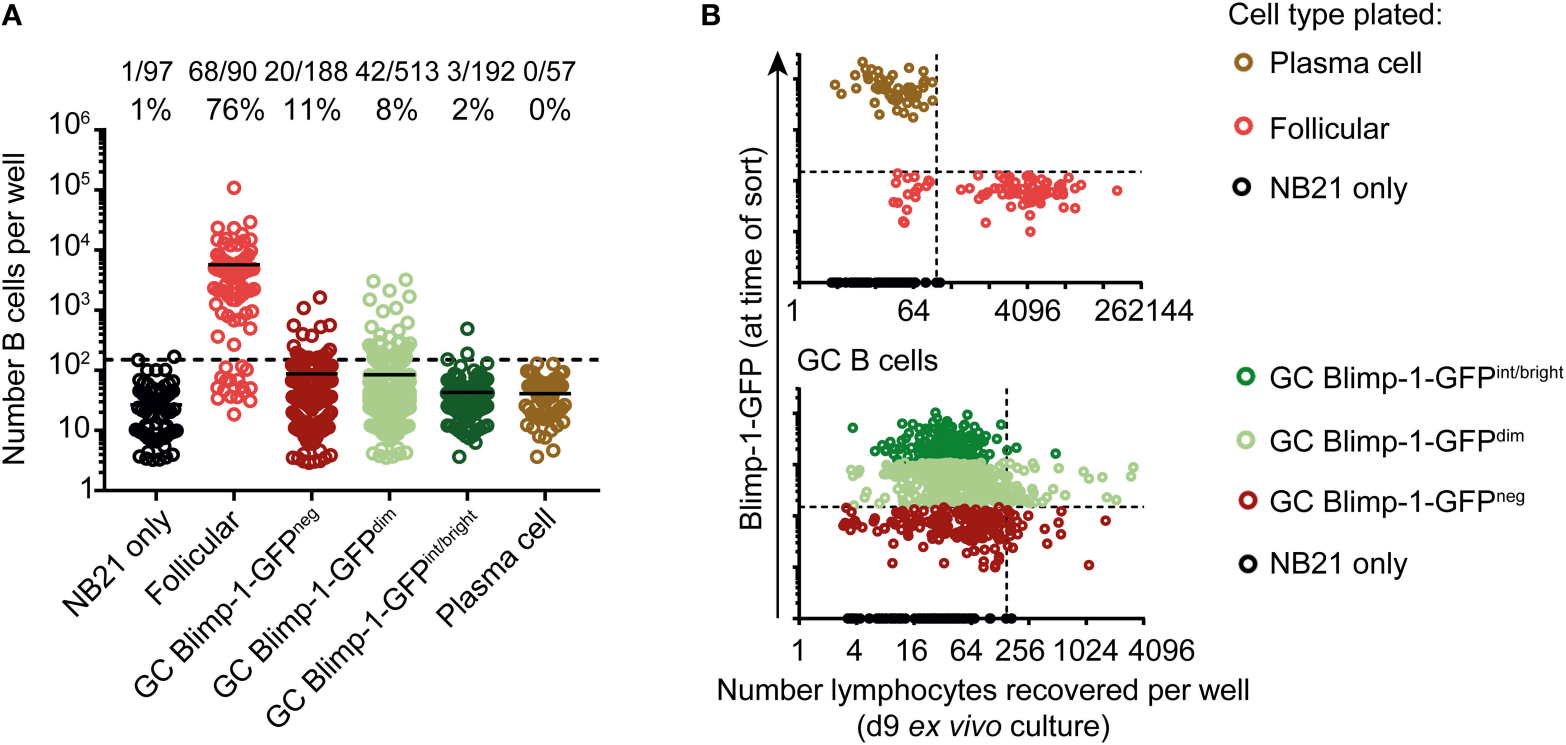
GC B cells expressing low Blimp-1 levels retain potential for extensive clonal expansion. **(A)** Single splenic GC B cells (B220^+^ IgD^low^ CD38^neg^ GL7^+^) from *Prdm1*^*wt*/*gfp*^ mice were index sorted on day 10 after SRBC immunization and cultured *ex vivo* using the “Nojima culture” method in 96 well plates. Follicular B cells (B220^+^ IgD^+^ CD38^+^ GL7^neg^) and plasma cells (B220^int^ CD138^+^ Blimp-1-GFP^bright^) were also sorted, for comparison. **(A)** Clonal culture sizes were determined on culture day 9. Solid lines indicate means. Dashed lines indicate the detection limit set according to NB21 feeder cell only wells. **(B)** Cell numbers recovered after culture were plotted against Blimp-1-GFP level at time of the sort (index FACs data). Vertical dashed lines indicate the detection limit. Blimp-1-GFP detection threshold (horizontal dashed line) was set based on a GFP^neg^ control. GFP intensities gates in **(A)** were set according to the color code in **(B)**. Pooled data from two independent experiments are shown.

Cultures seeded from single B cells were enumerated to determine population sizes, with “NB21 cell only” wells being used to set detection thresholds. As expected, this approach was highly efficient for cloning follicular B cells, and moderately so for non-Blimp-1 expressing GC B cells (62 and 11%, respectively) (Figure 4A). Surprisingly, the cloning efficiency was similar for Blimp-1-GFP^neg^ and Blimp-1-GFP^dim^ GC B cells (11 vs. 8%), and the population sizes established were approximately the same. Therefore, GC B cells expressing Blimp-1 at low levels have not yet reached a differentiation stage that precludes significant clonal expansion. In contrast, we failed using this assay to expand GC B cells that were Blimp-1-GFP^int/bright^ (2% of cells cloned), indicating that they are probably already committed to terminal differentiation. Similar findings of no growth were made when “mature” plasma cells (GL7^neg^ B220^int^ CD138^+^ Blimp-1-GFP^+^) were cultured, as expected. A negative correlation existed between the intensity of the Blimp-1-GFP signal at the time of sorting and the potential of GC B cells for clonal expansion in this assay (Figure 4B) (Chi-square test, *p* = 0.0014 comparing Blimp-1-GFP^dim^ with Blimp-1-GFP^int/bright^). Although a positive cloning result in this assay does not prove a cell can or will proliferate *in vivo* in the GC, these findings do provide strong evidence that Blimp-1 is expressed at low levels by GC B cells without necessarily immediately impairing their capacity for further clonal expansion.

### T_fh_ Cells Are Not Acutely Required for Blimp-1 Expression by GC B Cells

**Figure 5:**
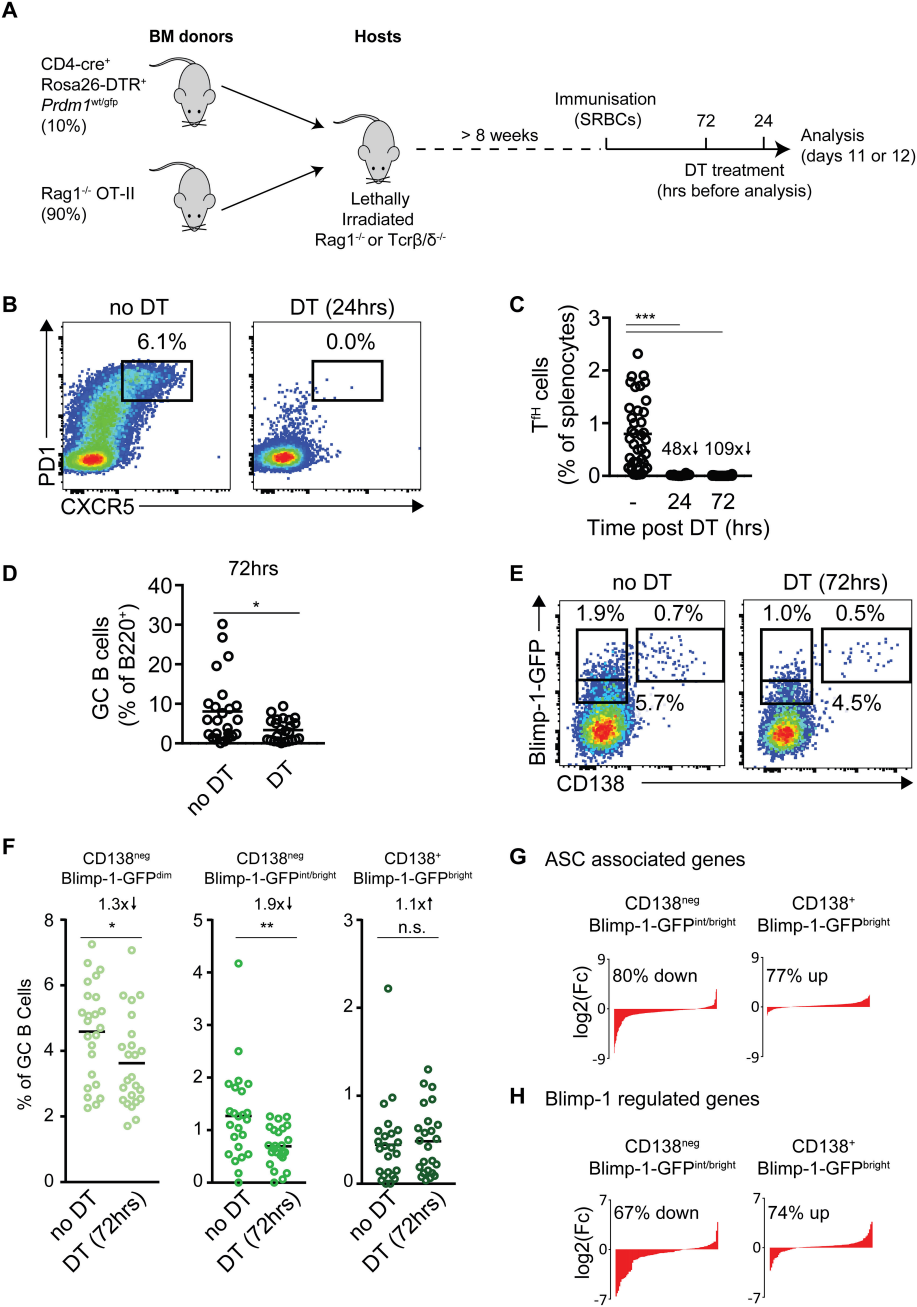
Blimp-1 expression by GC B cells is not acutely dependent upon cues from T cells. **(A)** Strategy for the temporal ablation of antigen specific T cells during an ongoing response. Lethally irradiated *Rag1*^−/−^ or TCR β/δ^−/−^ mice were reconstituted with the indicated mixes of bone marrow, then immunized with SRBCs >8 weeks later. Mice received single i.p. injections of diphtheria toxin (DT) 72 or 24 h before analysis on days 11 or 12. **(B)** CXCR5^+^ PD-1^+^ T_fh_ cells were identified, with and without 24 h DT treatment. Plots are gated on splenic B220^neg^ CD4^+^ cells and are from individual representative animals. **(C)** Frequencies of Tfh cells (as a proportion of splenocytes) and, **(D)** frequencies of GC B cells (as a proportion of total B cells), at the indicated time points post-DT treatment. **(E)** Representative CD138 vs. Blimp-1-GFP staining patterns on IgD^low^ CD95^+^ GL7^+^ GC B cells, 72 h after T cell ablation. **(F)** Summary of results as in **(E)**, from multiple mice/experiments. **(G,H)** RNA-seq analysis was performed on the indicated GC B cell populations from mice with and without DT treatment (72 h). **(G)** Changes in expression of ASC-related and **(H)** Blimp-1 activated genes in indicated GC B cell populations were determined and sorted by log2 fold differences in DT treated vs. control mice. Numbers indicate the proportion and direction of ASC or Blimp-1 regulated genes that are changed in treated mice. For **(C)** no DT *n* = 38, 24 h DT *n* = 10 or 72 h DT *n* = 24 mice. For all populations of **(D,F)** *n* = 24 mice from 5 independent experiments. For **(C,D,F)** a Kruskal-Wallis test with Dunn's test was performed. Horizontal lines indicate means. *P < 0.05; **P < 0.01; ***P < 0.001.

To directly test whether T_fh_ cells drive Blimp-1 expression as an alternative fate to cyclic re-entry, we established an experimental system in which antigen-specific T cells could be acutely ablated at defined periods after GCs had formed. *Rag1*^−/−^ or TCRβ/δ^−/−^ mice were lethally irradiated and reconstituted with mixes of bone marrow (BM) from *Rag1*^−/−^ OT-II mice and from CD4-Cre^+^ Rosa26-loxP-stp-loxP-DTR^+^ *Prdm1*^wt/gfp^ animals, at ratios of ~90:10 (Figure 5A). In the resulting bone marrow chimeric animals, all CD4^+^ T cells that are capable of responding to SRBC immunization express the human diphtheria toxin receptor (DTR) that allows their specific and temporal ablation by delivery of diphtheria toxin (DT). The co-transfer of *Rag1*^−/−^ OT-II BM cells provides a source of “irrelevant” non-ablatable hen egg ovalbumin-specific CD4^+^ T cells that will not respond to the immunizing antigen, and also prevents the complete depletion of CD4-expressing non-T cell lineages such as dendritic cells. Importantly, all B cells in the resulting chimeric animals carry the *Prdm1*^*gfp*^ allele but do not express DTR.

Cohorts of mixed BM chimeric mice were immunized with SRBCs and treated with DT at 24 or 72 h before tissue harvest on days 11 or 12 (Figure 5A). An analysis of the frequency of splenic CXCR5^high^ PD1^high^ T_fh_ cell frequencies confirmed DT mediated T cell ablation was fast and efficient, with 48- and 109-fold decreases at the 24 and 72 h time points (Figures 5B,C). GC B cells were still present even 72 h after DT treatment, but at reduced frequencies (Figure 5D). Strikingly, despite the near complete absence of cognate T cells for at least 2 days, Blimp-1-GFP expression was still detected in appreciable frequencies of the GC B cell populations at the 72 h time point (Figures 5E,F). Furthermore, the full range of “early,” “mid,” and “late” (CD138^neg^ Blimp-1-GFP^dim^, CD138^neg^ Blimp-1-GFP^int/bright^ and CD138^+^ Blimp-1-GFP^bright^) subsets were present, albeit at slightly reduced frequencies for “early” and “mid” stages (1.3 × and 1.9 × reduced, respectively). The least affected Blimp-1-GFP^+^ GC subset was the “latest” CD138^+^ Blimp-1-GFP^bright^ stage, which was reduced numerically but not in frequency. While these experiments using complex BM settings suffered from significant experiment to experiment variation in terms of total Blimp-1-GFP^+^ frequencies, similar results in terms of patterns of changes were noted in all experiments performed (Figures S4A,B). Furthermore, blocking CD40-CD40L signaling through delivery of the MR-1 anti-CD40L monoclonal antibody lead to similar effects in terms reductions in GC B cell size and frequencies of Blimp-1-GFP^+^ subsets (but with Blimp-1-GFP still detectable in appreciable fractions of cells) (Figures S4B–D).

To try to gain further insight into how cues provided by T cells may impact GC to pre-plasma cell differentiation, the transcriptomes of “mid” CD138^neg^ Blimp-1-GFP^int/bright^ and “late” CD138^+^ Blimp-1-GFP^bright^ cells, from mice that did or did not receive DT treatment 72 h earlier, were determined by RNA-seq. We focused on these subsets because they displayed stronger ASC-like gene expression signatures in earlier experiments (Figure 2). Consistent with CD138^neg^ subsets being the most impacted in terms of population frequency decreases after DT treatment (Figures 5E,F), the CD138^neg^ Blimp-1-GFP^int/bright^ subset was also the most affected in terms of numbers of genes whose expression was different after DT treatment (Figure S4E and Table S2). More specifically, the expression of many ASC-associated and Blimp-1 activated genes were reduced in this subset when T cells were ablated, suggesting that their differentiation may be delayed or stunted (Figures 5G,H and Table S2). The opposite trend was seen for the CD138^+^ subset where expression of Blimp-1-activated and ASC-associated genes was seemingly increased in mice that received DT. This may reflect that GC B cells less frequently reach this stage when T cells are not present, and as such this gate becomes enriched for cells that have been differentiating and accumulating in the gate for longer time periods.

Together, these findings indicate that T cell derived selection cues are not acutely needed for Blimp-1 expression by GC B cells, but that T cells promote normal numbers of Blimp-1-expressing GC B cells. The observed changes in gene expression of Blimp-1 expressing cells from DT treated animals suggests the possibility that T cells may also promote late stages of the pre-PC differentiation program.

### Dark Zone Cells Expressing Blimp-1 at Low Levels Can Reverse Expression *ex vivo* but May Be Sensitized for Acquiring Plasma Cell Characteristics

The findings that CD138^neg^ Blimp-1-GFP^dim^ GC B cells have transcriptional signatures very close to that of non-Blimp-1-expressing GC B cells, and that they retained a capacity for extensive proliferation *ex vivo*, raised the possibility that these “early” cells may not yet be fully committed to become antibody secreting cells. We therefore wished to directly test whether GC B cells with low Blimp-1 levels demonstrate differences in their propensity for further differentiation. To this end, we again used the “Nojima culture” protocol, but this time used it to support and stimulate small populations of GC B cells over 2 days *ex vivo* culture periods. These experiments were performed using mice expressing a *Bcl2*-transgene (57), in addition to the *Prdm1*^*gpf*^ allele, in order to enhance cell survival. Population sizes of 500 B cells were chosen as a compromise between feasibility of FACS sorting very rare subsets and recovering sufficient cell numbers for analysis at day 2.

**Figure 6:**
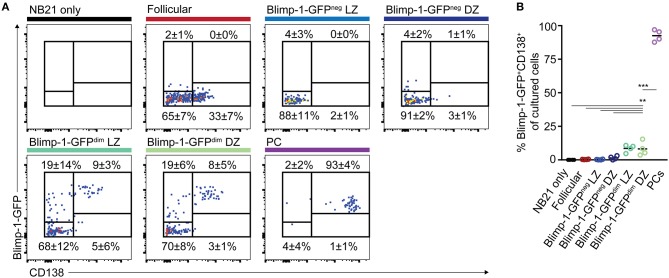
“Early” CD138^neg^ Blimp-1-GFP^dim^ cells may be primed to become plasma cells but the differentiation program is not yet hard set. **(A)** Bcl2-tg^+^ *Prdm1*^wt/gfp^ mice were immunized with SRBCs and the indicated splenic GC B cell populations (CD38^neg^ IgD^neg^ GL7^+^) were sorted on day 10 (500 cells/condition). CD138^neg^ Blimp-1-GFP^dim^ and LZ/DZ gates were set using the scheme outlined in Figures 1B,D, but with slightly increased stringency to ensure purity. Follicular B cells (IgD^+^CD38^+^GL7^neg^) and plasma cells (B220^int^ CD138^+^ Blimp-1-GFP^bright^) were also sorted, for comparison. **(A)** Sorted cells were cultured under “Nojima” conditions for 48 h, before analysis for Blimp-1-GFP and CD138 expression. Numbers indicate the frequencies of cells which were positive or negative for the respective markers. **(B)** Data from multiple experiments are summarized. Dot colors relate to titles on left. Data is pooled from 4 independent experiments with one animal sorted per experiment. A one-way ANOVA with Dunnett's test was performed **P < 0.01; ***P < 0.001.

In cultures where B cells did not express Blimp-1-GFP at the time of plating, most cells remained GFP^neg^ at the time of analysis, with only 3.8% (LZ) and 5.9% (DZ) of the cells becoming GFP^+^ (Figures 6A,B). As expected, cultured plasma cells mostly remained Blimp-1-GFP^+^ and CD138^high^. Somewhat surprisingly, many of the CD138^neg^ Blimp-1-GFP^dim^ cells (both of DZ and LZ phenotype) lost detectable GFP expression by the time of analysis, indicating that Blimp-1 expression had been reversed and that terminal plasma cell commitment was not yet hard set. Whether this might be the explanation for why CD138^neg^ Blimp-1-GFP^dim^ cells and non-Blimp-1 expressing cells expanded equivalently well in the 9 day single cell *ex vivo* assays (Figure 4), or whether instead this resulted from continued expansion by cells that maintained Blimp-1 expression but are not yet fully differentiated, is not clear. Despite Blimp-1 reversion by some cells, however, CD138^neg^ Blimp-1-GFP^dim^ populations did seemingly also contain within them cells that displayed evidence of being primed or sensitized for plasma cell differentiation, because just over one quarter of the cells remained GFP^+^ (27.1% for DZ cells and 27.3% for LZ), and small frequencies upregulated CD138 (8.2% for DZ cells and 8.5% for LZ). CD138 levels on cultured GC B cells were lower than on cultured plasma cells, however its expression was mostly restricted to Blimp-1-GFP^brighter^ cells supporting it being differentiation associated. We also performed ELISA assays on the culture supernatants to ask whether antibody secretion had occurred, however results from these experiments were variable and as such inconclusive (not shown).

Together, these results indicate that CD138^neg^ Blimp-1-GFP^dim^ GC B cell populations are not yet committed to irreversible Blimp-1 expression but that they may contain within them cells that may be primed or sensitized to acquire further PC-associated characteristics.

## Discussion

We investigated the context in which Blimp-1 is expressed by GC B cells with the goal of better understanding the processes that drive their differentiation toward a plasma cell fate. Consistent with recent findings from Kräutler et al. (28), we report that Blimp-1 is expressed at low levels by a subset of LZ cells. More surprising, however, was the result that the largest of the “early” CD138^neg^ Blimp-1-GFP^dim^ subsets was one with a DZ phenotype. A trivial explanation for these results might have been that these cells increased their CXCR4 levels, and decreased surface CD83 and CD86 abundance, as a consequence of them becoming plasma cells rather than because they were bona fide DZ cells. However, this conclusion was not supported by transcriptome analysis that we performed. Moreover, while GC cells mostly exit cell cycle before entering the LZ, the behavior of Blimp-1-expressing DZ cells was similar to that of their Blimp-1^neg^ counterparts in that they were rapidly dividing. Therefore, Blimp-1 is expressed at low levels by proliferating cells engaged in the GC DZ program.

An association between proliferation and Blimp-1 induction has been recognized in other settings for many years. After mitogenic stimulation *in vitro*, as well as during plasmablast differentiation *in vivo*, cells undergo plasma cell differentiation only after completing multiple cell divisions (58–60). It was not possible for us to conclusively determine whether Blimp-1 expression was induced in, rather than maintained by, DZ cells, however our results do indicate an association between Blimp-1 transcription and a highly proliferative state. High affinity LZ cells receive cues that lead them to divide faster and more times upon returning to the DZ (11, 12), therefore the same cues that drive this proliferation might indirectly cause some daughter cells to acquire Blimp-1 competence. Such a pathway could contribute to the preferential development of higher affinity plasma cells. Interestingly, CD40L and IL-21, both of which are likely contributing inputs in terms of determining proliferation rates and numbers of division cycles by GC B cells, also have strong links to plasma cell differentiation (27, 61). Such a model might also help explain why, in experiments where T cell help is strongly augmented using a DEC205 scavenger receptor mediated peptide loading protocol (5), plasma cell development is enhanced without reducing GC participation.

How then might single selected GC B cells, after entering the DZ, generate Blimp-1^+^ and Blimp-1^neg^ progeny? Continuous live cell imaging and mathematical modeling approaches have suggested that disparate B cell fates may develop due to stochastic variation in how competition plays out between opposing components of the cell's signaling circuitry (62). If this were true in the GC, bifurcation would not necessarily need to occur immediately following a particular event such as antigen engagement or T cell interaction. Interestingly, even 2-fold changes in Bcl6 and Pax5 levels can impact GC B cell fate decisions (63). Alternatively, the adoption of different fates might involve the unequal inheritance of B cell and plasma cell promoting factors during mitosis (64). For example, asymmetrical distribution of BCR-Ag complexes, signaling units, Bcl6 or Blimp-1 repressing/enhancing factors could cause daughter cells to diverge in terms of how they respond to certain environmental cues or signaling events (65, 66).

An important finding from our work is that T cell derived signals are not acutely required for Blimp-1 expression in the GC. As such, T cells do not drive this fate as an alternative to cyclic re-entry. While these finding are consistent with those of Kräutler et al. who recently depleted T cells using an antibody-mediated approach (28), Ise et al. instead found that the generation of a LZ plasma cell precursor cells (defined as Bcl-6^low^ CD69^high^ IRF4^high^) required strong signals via the CD40-CD40L axis (27). An important unresolved issue, therefore, is what is the relationship between the two reported cell states? The subset of cells described in the Ise et al. study was not yet detectibly Blimp-1^+^, whereas *Irf4* transcript levels were not increased in any of the earliest Blimp-1^+^ cell types reported here, even when they had a LZ phenotype. Interestingly, Bcl-6^low^ CD69^high^ IRF4^high^ cells were found to also express *c-myc* and *Batf* at high levels, and these are both required for DZ maintenance (67–69). One possibility, therefore, is that the Bcl-6^low^ CD69^high^ IRF4^high^ subset contains cells that recently received strong selection cues and are destined to undergo proliferative bursts on their return to the DZ, which might indirectly lead to Blimp-1 expression.

While we have focused on understanding where and when Blimp-1 is expressed in the GC, whether or not its transcription alone (at least at low levels) exclusively marks cells that are destined to leave the GC is not clear. “Early” CD138^neg^ Blimp-1-GFP^dim^ cells, regardless of their DZ/LZ profile, maintained transcriptional signatures very close to that of “normal” GC B cells. Furthermore, 2 days after their isolation and co-culture under “Nojima” conditions, many CD138^neg^ Blimp-1-GFP^dim^ cells became GFP^neg^ again, indicating that Blimp-1 transcription had been transient. Although it is important to note that no culture conditions accurately mimic the *in vivo* setting, and as such it is not clear whether similar reversion occurs in GCs, these results do indicate that the plasma cell differentiation program is not yet hard set in these cells. Despite the Blimp-1 reversion by some cells, however, other cells from the same culture wells maintained Blimp-1 expression and acquired another plasma characteristic by upregulating CD138. Therefore, we speculate that low Blimp-1 expression in the GC may mark cells (or populations containing cells) that are not yet committed to PC differentiation, but are competent to do so.

Although help from T cells was not acutely required for Blimp-1 expression in our experiments, we do not exclude the possibility that cues received from them promote later PC commitment events. Kräutler et al. reported that, following antibody mediated depletion of T cells, plasma differentiation was halted at an immature Blimp-1-GFP^low^ stage (28). As such, it remains possible that T cells may contribute to affinity discrimination at late differentiation stages, or that T cells have one last say in terms of ensuring cells are of an appropriate specificity. We did not observe an absolute loss of any particular differentiation subset following T cell ablation in our experiments, however we did note that CD138^neg^ Blimp-1-GFP^int/bright^ cells were less developed in terms of their expression of ASC associated genes, which would be consistent with a late differentiation block. Whether these gene expression differences reflected a global impairment in differentiation, or instead indicated that fewer cells reached later stages, was not assessed. Somewhat surprisingly, “late” CD138^+^ Blimp-1-GFP^bright^ cells were the least affected subset by T cell ablation in our assays, but this might reflect that these cells persist for longer after they are generated. The concept that CD138^+^ GC B cells might remain in that state for relatively prolonged periods is seemingly supported with the finding by Laidlow et al. that these cells are negative for a fluorescent *S1pr2* transcriptional reporter (mVenus) that presumably has a half-life of many hours (70), despite our detecting S1pr2 transcript in “early” CD138^neg^ Blimp-1-GFP^dim^ and “mid” CD138^neg^ Blimp-1-GFP^int/bright^ cells. While we did not test a requirement for BCR mediated antigen engagement in supporting or promoting plasma cell differentiation (independently of peptide acquisition for presentation to T cells), Kräutler et al. showed that blocking antigen engagement leads to a near complete loss of Blimp-1 expression by GC B cells (28). Given that antigen is mostly found in the LZ, it is not immediately obvious how these results fit with our findings of Blimp-1 expression in the DZ. However, a plausible explanation might be that BCR signaling events received at earlier LZ stages contributes to Blimp-1 initiation some time later after cells have returned to the DZ. Further studies are required to resolve these important issues.

In summary, we find that GC B cells express the plasma cell master regulator Blimp-1 at low levels during periods of active proliferation in their DZ state. Blimp-1 expression in the GC does not necessarily commit cells to a plasma cell fate, but it probably does mark cells that are sensitized to going down this route. The correlation of proliferation and Blimp-1-induction suggests the possibility that the two may be linked, especially given the previously described associations in other settings. As such, progeny arising from single selected cells may, in principle, seed plasma cell populations without impairing their continued participation within the GC response.

## Data and Materials Availability

RNA-seq data is available at National Center for Biotechnology Information's Gene Expression Omnibus (GEO) under the GEO series accession number GSE111419. https://www.ncbi.nlm.nih.gov/geo/query/acc.cgi?acc=GSE111419

## Author Contributions

DR performed the experiments. DR and OB designed and analyzed the experiments and wrote the manuscript. OB supervised the project.

### Conflict of Interest Statement

The authors declare that the research was conducted in the absence of any commercial or financial relationships that could be construed as a potential conflict of interest.
