# Supplementary material for: Expression of the Plasma Cell Transcriptional Regulator Blimp-1 by Dark Zone Germinal Center B Cells During Periods of Proliferation
Source: Front Immunol. 2019 Jan 9;9:3106. doi: 10.3389/fimmu.2018.03106 (PMC6334666; doi:10.3389/fimmu.2018.03106)
Supplement: Supplementary file 4 [file Data_Sheet_1.docx]

Supplementary Material

Expression of the plasma cell transcriptional regulator Blimp-1 by germinal center dark zone cells during periods of proliferation.

Daniel Radtke, & Oliver Bannard*

*** Correspondence:** Oliver Bannard: oliver.bannard@ndm.ox.ac.uk

**Supplementary Materials:**

**Suppl. figure 1. A Blimp-1-GFP^dim^ population is present in the germinal center dark zone (Related to Fig. 1).** *Prdm1^wt/gfp^* mice were immunised with SRBCs and splenic GC B cells were analysed on day 10. (A) Blimp-1-GFP^neg^ cells or Blimp-1-GFP^pos^ GC B cells were gated as in Fig 1A. Colored dots are backgated cells superimposed on total B cell populations, with GC gates shown. (B) Blimp-1-GFP^+^ cells were sub-divided based upon GFP and CD138 levels, shown to left. The frequency of LZ and DZ cells within each gate was determined using a CXCR4/CD86 gating scheme. (C-F) Similar experiments were performed using *Prdm1*-Venus BAC transgenic (C, D), and *Prdm1*-YFP BAC transgenic (E, F), mouse lines. (D) and (F) show total GC B cells. The dashed lines indicates fluorescence detection limit. (G) *Prdm1^wt/gfp^* mice were infected with HKx/31 influenza A virus and similar analysis was performed on mediastinal lymph node GC B cells, on day 11. (H) CXCR4 and Blimp-1-GFP levels on total GC B cells from the influenza infections. (I, J) Similar analysis to that in (B) was performed, but using alternative CD95^+^ PNA^+^ (I) and CD95^+^ EphrinB1^+^ (J) GC B cell gates. (K) Additional examples of splenic GCs from Prdm1^wt/gfp^ mice in which Blimp-1-GFP^+^ cells were identified. Not all GCs contained detectable GFP^+^ cells, but GCs without them are not shown. Data in (B) are representative of 8 mice from 2 experiments, (C) and (D) are representative of 6 mice from 2 experiments, (E) and (F) are representative of 9 mice from 3 experiments. (G) and (H) are representative of 9 mice from 2 experiments. (I) is representative of 7 mice from 2 experiments. (J) is representative of 12 mice from 3 experiments. (K) germinal center sections from 5 mice for experiment 1 and 6 mice for experiment 2. Numbers indicate percent gated ± S.D..

**Suppl. figure 2. GFP^+^ LZ B cells retain transcriptomes consistent with them being an early differentiation stage arising from that zone (Related to Fig. 2).** (A) Relative expression level of LZ signature genes, with examples discussed in the text highlighted in red. (B) Venn diagrams displaying numbers of gene expression changes detected in each of the CD138^neg^ Blimp-GFP^dim^ subsets, relative to their non-Blimp-1-GFP expressing counterparts (adj. p-value <0.05, RPKM >5). Numbers of genes with expression changes detected in the later CD138^neg^ Blimp-1-GFP^int/bright^ subsets are shown also. Gene expression changes apparent in both the LZ and DZ subsets are indicated in the lower Venn diagram. Heatmaps indicate the relative changes in expression of genes identified as differing between DZ^neg^ vs DZ^dim^ and DZ^int/bright^ (left) and LZ^neg^ vs LZ^dim^ LZ^int/bright^ (right). Also shown are genes whose expression differs in all CD138^neg^ Blimp-1-GFP LZ and DZ subsets (i.e. those increased or decreased in CD138^neg^ Blimp-GFP^dim^ and CD138^neg^ Blimp-GFP^int/bright^ LZ and DZ cells) (center). Blue/green boxes to left indicate genes reported as being ASC associated (blue) or commonly changed in during late stages of antibody secreting cell development (green) (Shi et al. 2015).

**Suppl. figure 3. CD138^neg^ Blimp-1-GFP^dim^ GC B cells are actively dividing (Related to Fig. 3).** (A) DNA content (DAPI staining) was determined for splenic GC B cells (CD95^+^ GL7^+^) with high and low CXCR4 levels, on day 10 post SRBCs immunisation. The dashed line (left plot) indicates GFP detection threshold based upon non-fluorescent wild-type samples. Plots to right are gated on Blimp-1-GFP^neg^ and Blimp-1-GFP^+^ cells. (B, C) Similar assessments as in Fig. 3B were performed on IgD^low^ CD95^+^ GL7^+^ GC B cells at days 6 (B) and 16 (C) post- SRBC immunisation. (D) Additional examples of confocal images in which Blimp-1-GFP^+^, EdU^+^ double positive cells were detected in the GCs. Double positive cells are highlighted by orange rectangles and are shown in magnification with or without GFP or EdU channel to side. For (B, C) a Kruskal-Wallis test with Dunn’s test was performed (n=8; pooled from 3 independent experiments). Horizontal lines indicate means *, P < 0.05; **, < 0.01; ***, P < 0.001.

**Suppl. Figure 4. Blimp-1 expression by GC B cells is not acutely dependent upon cues from T cells (Related to Fig. 5).** (A) Results from individual T cell ablation experiments, with each dataset plotted separately. Graphs show frequency of indicated subsets with or without DT treatment 72hrs earlier. (B) *Prdm1^gfp/+^* mice were immunised with SRBCs and then treated with anti-CD40L (clone MR-1, 0.5mg/mouse), or with an isotype control antibody, 48hrs before analysis. Frequencies of GC B cells (among B220^+^ cells) are shown. C) Representative CD138 vs Blimp-1-GFP plots, with means ± S.D.. (D) Summary of results from multiple mice and experiments. (E) Experimental setup was performed as in Fig. 5A. RNAseq analysis was performed on the indicated subsets 72 hrs after DT treatment. Log_2_ RPKM values indicating the numbers of genes whose expression is increased or decreased when T cells have been ablated. Red dots mark genes that are statistically significant differentially expressed (adj. p-value <0.05, fold change >2.5, RPKM >5 in at least one condition).

**Additional files:**

**Table S1.** RPKM values, gene set data and differential expression data for Blimp-1-GFP^+^ and Blimp-1-GFP^neg^ populations (Related to Fig. 2)

**Table S2.** RPKM values, Gene set data and differential expression data for T cell ablation experiment (Related to Fig. 5)

**Table S3.** List of reagents, antibodies and gene expression assays.
