# Supplementary figures and images for: Expression of the Plasma Cell Transcriptional Regulator Blimp-1 by Dark Zone Germinal Center B Cells During Periods of Proliferation
Source: Front Immunol. 2019 Jan 9;9:3106. doi: 10.3389/fimmu.2018.03106 (PMC6334666; doi:10.3389/fimmu.2018.03106)

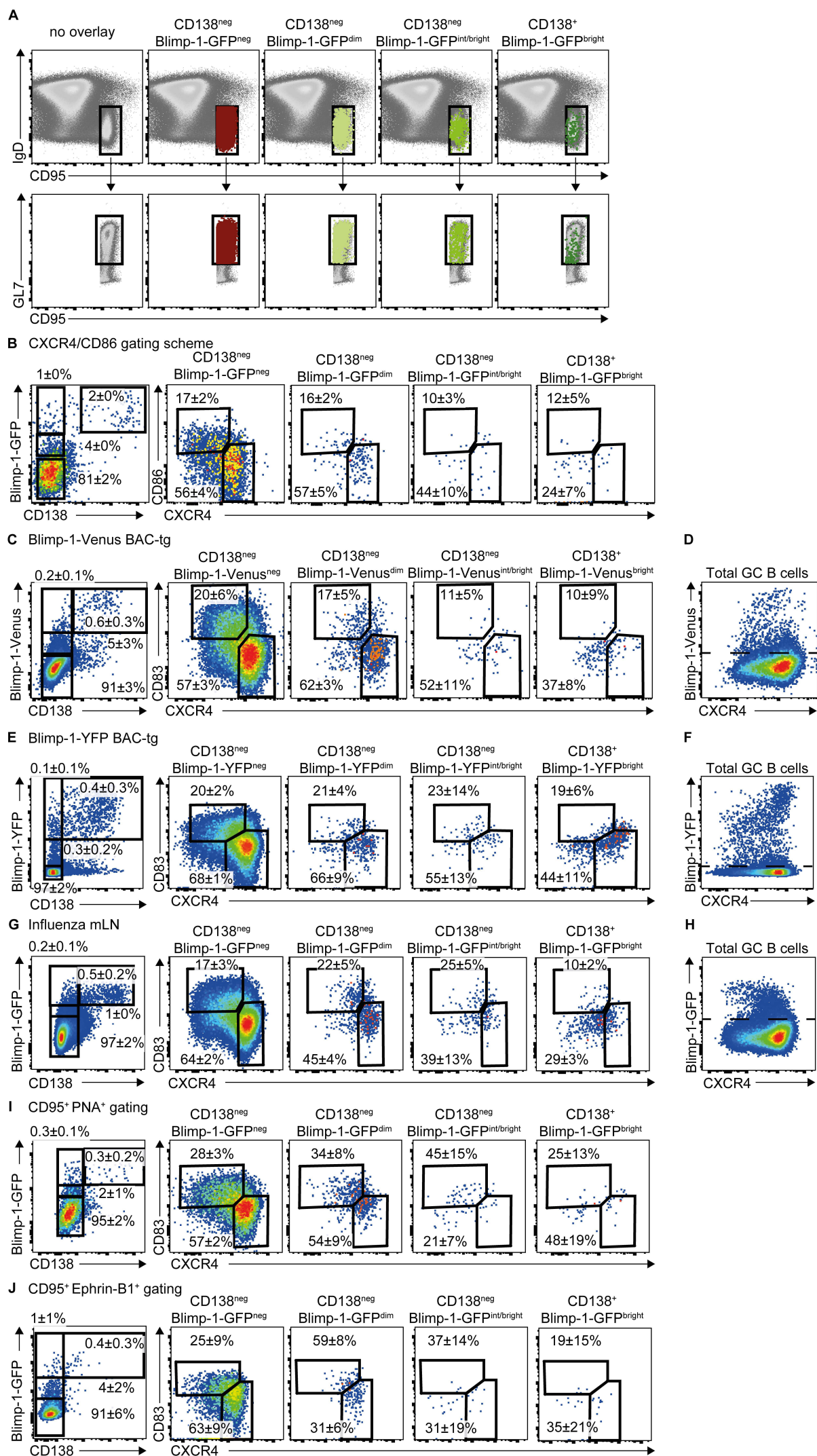

**Fig. S1**  
**Page 1**

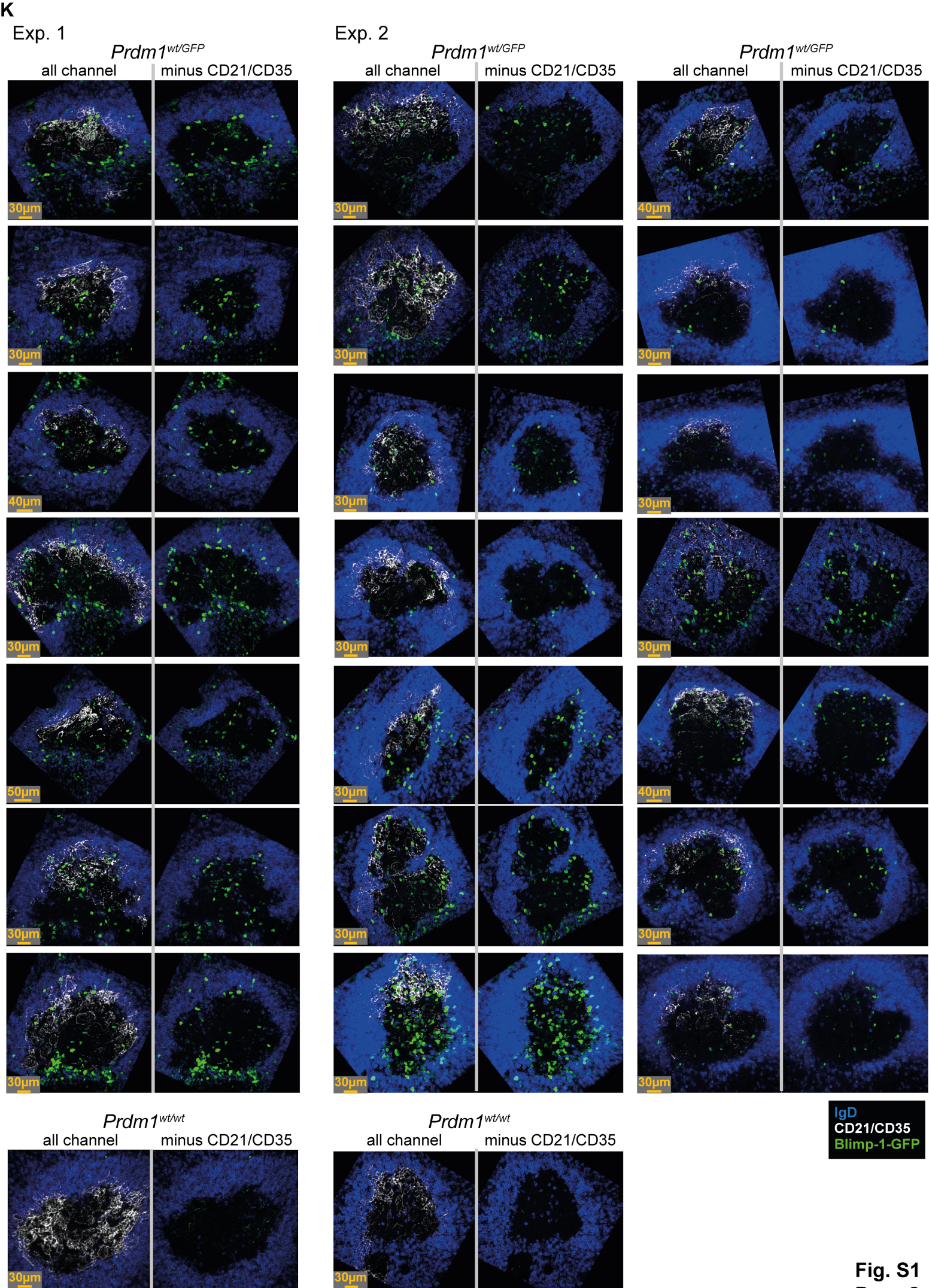

**Fig. S1**  
**Page 2**

Supplement: Supplementary file 5 [file Image_1.pdf]

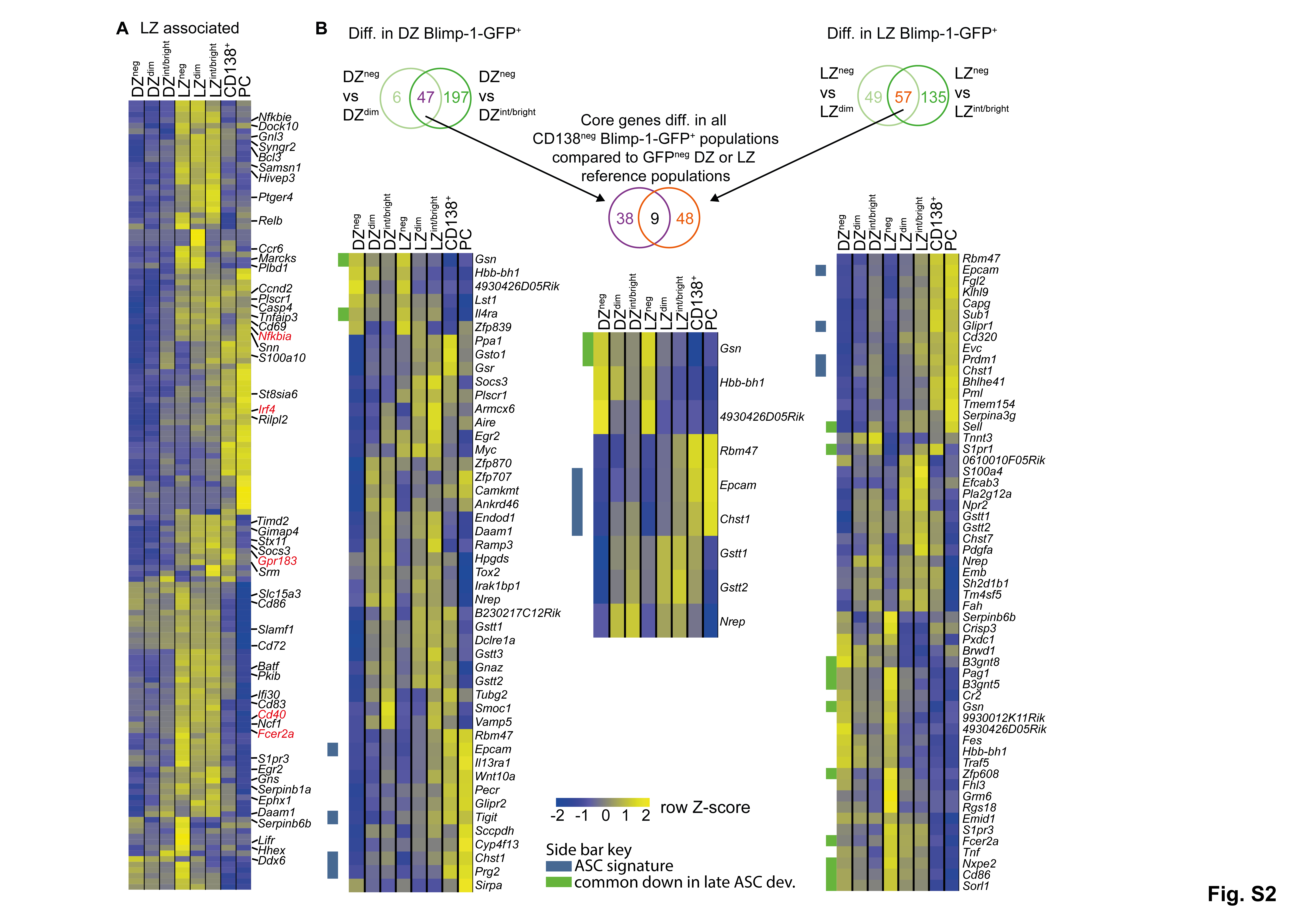

Supplement: Supplementary file 6 [file Image_2.tif]

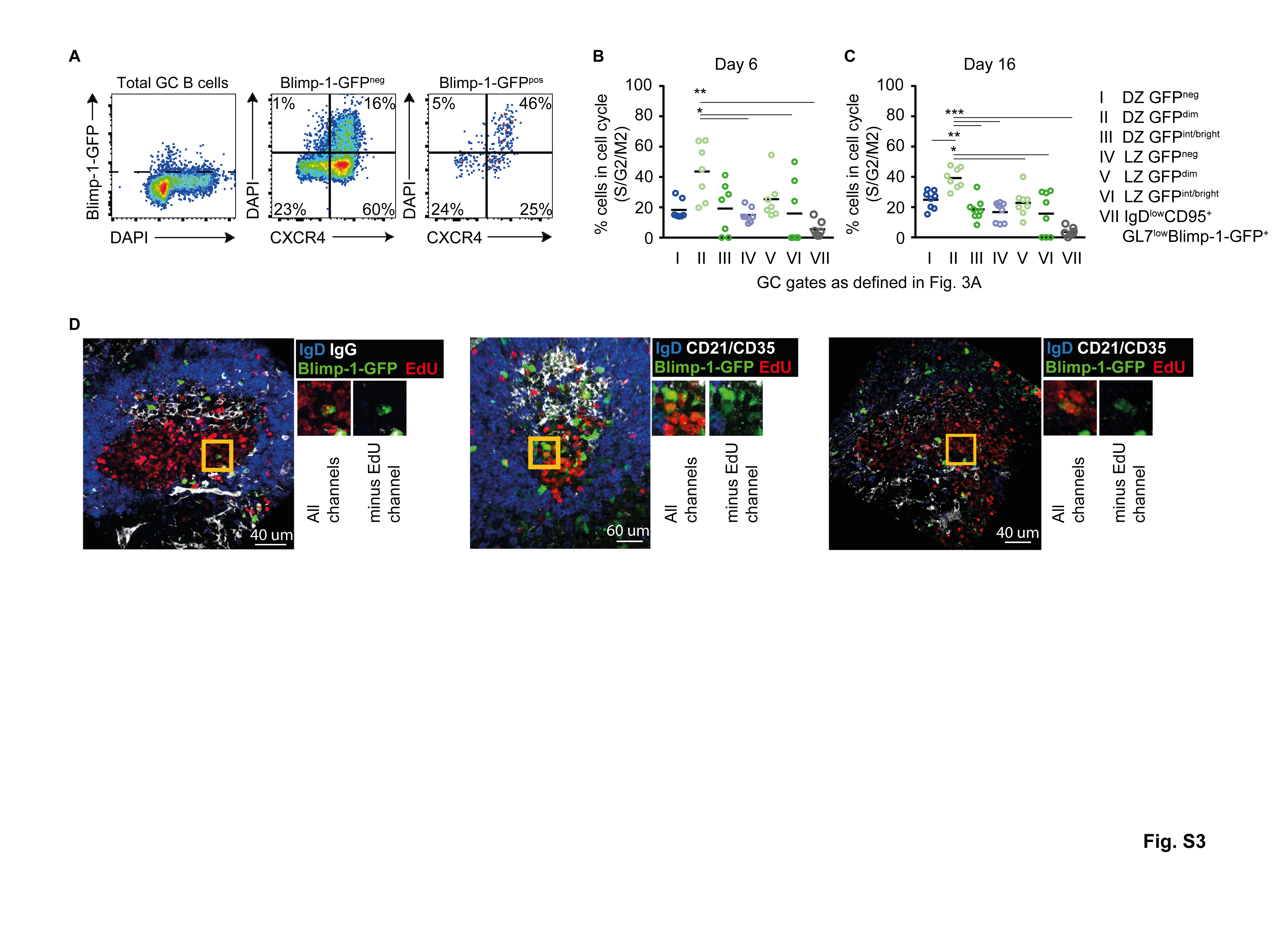

Supplement: Supplementary file 7 [file Image_3.tif]

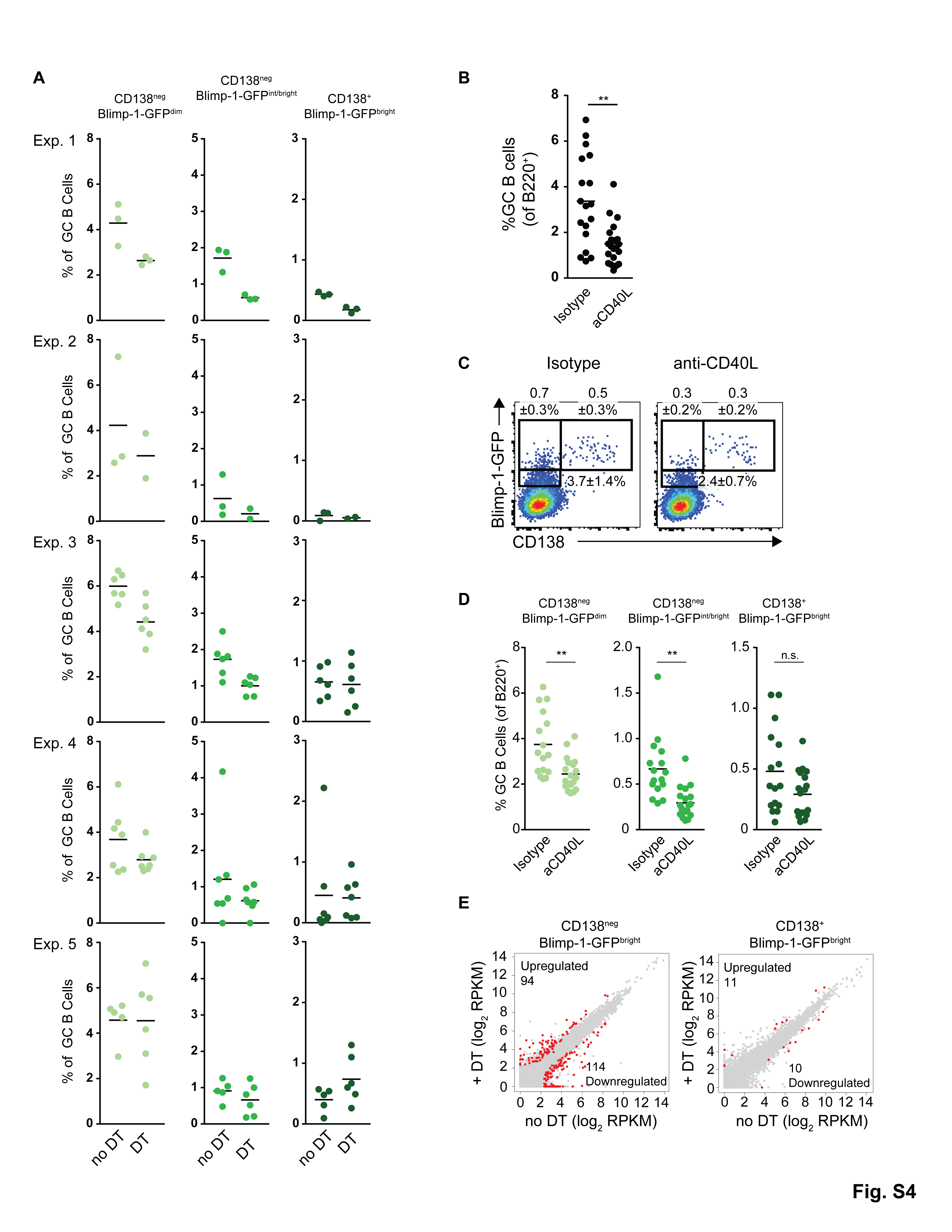

Supplement: Supplementary file 8 [file Image_4.tif]
